# Photoacoustic Computed Tomography of Breast Cancer in Response to Neoadjuvant Chemotherapy

**DOI:** 10.1002/advs.202003396

**Published:** 2021-02-23

**Authors:** Li Lin, Xin Tong, Peng Hu, Marta Invernizzi, Lily Lai, Lihong V. Wang

**Affiliations:** ^1^ Caltech Optical Imaging Laboratory Andrew and Peggy Cherng Department of Medical Engineering California Institute of Technology Pasadena CA 91125 USA; ^2^ Division of Surgical Oncology Department of Surgery City of Hope Comprehensive Cancer Center Duarte CA 91010 USA

**Keywords:** breast cancer, neoadjuvant chemotherapy, photoacoustic computed tomography, response to treatment, tumor‐associated microvasculature

## Abstract

Neoadjuvant chemotherapy (NAC) has contributed to improving breast cancer outcomes, and it would ideally reduce the need for definitive breast surgery in patients who have no residual cancer after NAC treatment. However, there is no reliable noninvasive imaging modality accepted as the routine method to assess response to NAC. Because of the inability to detect complete response, post‐NAC surgery remains the standard of care. To overcome this limitation, a single‐breath‐hold photoacoustic computed tomography (SBH‐PACT) system is developed to provide contrast similar to that of contrast‐enhanced magnetic resonance imaging, but with much higher spatial and temporal resolution and without injection of contrast chemicals. SBH‐PACT images breast cancer patients at three time points: before, during, and after NAC. The analysis of tumor size, blood vascular density, and irregularity in the distribution and morphology of the blood vessels on SBH‐PACT accurately identifies response to NAC as confirmed by the histopathological diagnosis. SBH‐PACT shows its near‐term potential as a diagnostic tool for assessing breast cancer response to systemic treatment by noninvasively measuring the changes in cancer‐associated angiogenesis. Further development of SBH‐PACT may also enable serial imaging, rather than the use of current invasive biopsies, to diagnose and follow indeterminate breast lesions.

## Graphical abstract

A photoacoustic imaging system is developed to noninvasively assess breast cancer's response to neoadjuvant chemotherapy (NAC) by imaging breast cancer patients before, during, and after therapy. The analysis of tumor size, blood vascular density, and irregularity in the distribution and morphology of the blood vessels accurately identifies cancer's response to NAC as confirmed by the histopathological diagnosis.

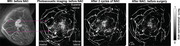

## 1 Introduction

Although breast cancer remains the second leading cause of female cancer deaths in the United States, significant progress has been made in improving overall survival.^[^ ^1^ ^]^ The development and increased use of effective and directed systemic therapy based on biological characterization of the subtypes of breast cancer have directly contributed to improvement in survival outcomes.^[^ ^2^, ^3^, ^4^ ^]^ Specifically, neoadjuvant chemotherapy (NAC), systemic therapy that is given before surgery, has contributed to improved cancer outcomes in breast cancer patients by increasing the likelihood of breast conservation^[^ ^5^, ^6^ ^]^ and by providing patients with important prognostic information based on response to treatment.^[^ ^7^, ^8^ ^]^ Confirmation of cancer response to NAC is associated with improved disease‐free and overall survival.^[^ ^8^ ^]^ In addition, evaluation of breast cancer response to NAC assists in drug development.^[^ ^9^, ^10^ ^]^ Lastly, exceptional responders to NAC may have eradication of their breast cancers. In these exceptional responders, the risk of breast cancer recurrence is low with associated excellent 5‐year relapse free survival of 95% and 94% in patients with HER2+ and Triple Negative breast cancers, respectively.^[^ ^11^ ^]^ These findings argue against the need for definitive surgery and directly challenge the current paradigm of surgical resection established for solid tumors. Indeed, interest in the elimination of surgery in patients with exceptional response has resulted in active prospective clinical trials to evaluate the outcomes in women who receive NAC and do not undergo definitive surgery.^[^ ^12^ ^]^

At present, there is no reliable noninvasive imaging modality clinically accepted to determine complete cancer response to NAC. Consequently, response of the cancer to treatment is diagnosed only after the breast tissue is surgically removed. Current noninvasive clinical methods to monitor cancer's response to NAC include clinical breast examination, mammography, ultrasonography, and magnetic resonance imaging (MRI). These methods depend on cancer morphology and size measurements, which may not change despite the cancer's response to therapy. Furthermore, therapy‐induced fibrosis often impedes these modalities from accurately monitoring response to NAC.^[^ ^13^ ^]^

The ability to identify the optimal time point to differentiate responders from non‐responders would contribute greatly to clinical management of the breast cancer patients. Recent studies have begun to examine the functional and anatomical changes in cancer to improve the ability to assess response to therapy. These studies utilized fluorothymidine positron emission tomography (PET),^[^ ^14^ ^]^ contrast‐enhanced MRI (CE‐MRI) with vascular enhancement,^[^ ^15^ ^]^ and diffuse optical imaging (DOI).^[^ ^16^ ^]^ The early results of these investigational imaging methods highlight the potential for metabolic and hemodynamic measurements. However, both PET and CE‐MRI require the use of exogenous contrast agents and local facilities to either handle radioactive materials or a strong magnetic environment. These issues, as well as the duration of the imaging studies, are barriers against frequent and timely evaluations of patients treated with NAC. DOI can provide frequent label‐free monitoring of patients. However, the spatial resolution of DOI is limited,^[^ ^17^ ^]^ diminishing the potential of this modality for imminent clinical use. Therefore, there remains a critical need to develop imaging technology to improve on the assessment of breast cancer treated with NAC.

Photoacoustic computed tomography (PACT) combines the functional optical contrast of DOI and the high spatial resolution of ultrasonography, without speckle artifacts.^[^ ^18^ ^]^ The rich contrast provided by optical absorption, which is related to various intrinsic and extrinsic contrast origins, enables PACT to perform structural, functional, and molecular imaging.^[^ ^19^ ^]^ When a short‐pulsed laser irradiates biological tissues safely, wideband ultrasonic waves (referred to as photoacoustic waves) are induced as a result of transient thermoelastic expansion. The photoacoustic (PA) waves are then measured by ultrasonic transducers around the tissue and are used to reconstruct the optical absorption distribution in the tissue. At 1064 nm, which is the fundamental wavelength of Nd:YAG lasers, the 1/e optical attenuation coefficient for an average breast is around 0.9 cm^−1^,^[^ ^20^ ^]^ which is slightly higher than that for X‐rays (0.5–0.8 cm^−1^).^[^ ^21^ ^]^ However, the optical contrast of soft tissue is much higher than X‐ray contrast.^[^ ^22^ ^]^ For breast imaging, preliminary studies have shown that PACT can exploit its advantages to the fullest, offering high spatial and temporal resolution at sufficient depths,^[^ ^23^, ^24^ ^]^ using nonionizing radiation.

The use of PACT to assess response to NAC in breast cancer has been reported.^[^ ^25^, ^26^, ^27^ ^]^ However, previous PACT studies were unable to image detailed angiographic structures (i.e., individual blood vessels) due to system limitations such as insufficient spatial sampling rate and limited detection angle, thus limiting their accuracy and reliability. For example, the breast could be deformed differently during each imaging session, affecting the light fluence in the tissue to be inconsistent and unpredictable. As such, without the ability to reveal detailed breast anatomy, the quantitative measurements (e.g., total hemoglobin concentration) requiring prior knowledge of the optical distribution are unreliable. Recently, we developed a single‐breath‐hold PACT (SBH‐PACT) system capable of detailing angiographic structures of the whole breast within a single breath hold of 15 s. The imaging completed by SBH‐PACT clearly delineates tumor angiogenesis,^[^ ^23^ ^]^ a process that has been described as a hallmark of cancer growth and metastasis.^[^ ^28^, ^29^, ^30^ ^]^ In addition, an increase in vascular endothelial growth factor expression has been associated with impaired response to chemotherapy.^[^ ^31^, ^32^ ^]^ Taken together, the ability to image and analyze angiogenesis has the potential to detect changes in the breast cancer from disease progression or from response to treatment. In addition, since angiogenesis precedes transformation of mammary hyperplasia to malignancy, monitoring tumor‐associated microvasculature change over time may assist in early detection and diagnosis of breast cancer in patients with abnormal mammograms. Use of serial noninvasive imaging such as SBH‐PACT may lead to a more streamlined and accurate workup of indeterminate lesions and obviate the need for invasive diagnostic biopsies.

To better assess the ability of SBH‐PACT to determine breast cancer response to NAC, we imaged three breast cancer patients treated with NAC at three time points: before, during, and after NAC. We segmented the boundaries of the cancers and quantified the changes in cancer size and the density, entropy, and morphological irregularity of the cancer blood vessels. These analyses from the SBH‐PACT images were compared with the histopathological results, treated as ground truth, of the resected breast specimen. In this report, we describe our initial experience with SBH‐PACT and demonstrate that SBH‐PACT, comparable to a contrast‐free, high‐speed, and high‐resolution version of CE‐MRI, is capable of providing details and capturing changes associated with response of breast cancer to NAC.

## 2 Results

### 2.1 Evaluation of the SBH‐PACT System with a Breast‐Mimicking Phantom

**Figure 1:**
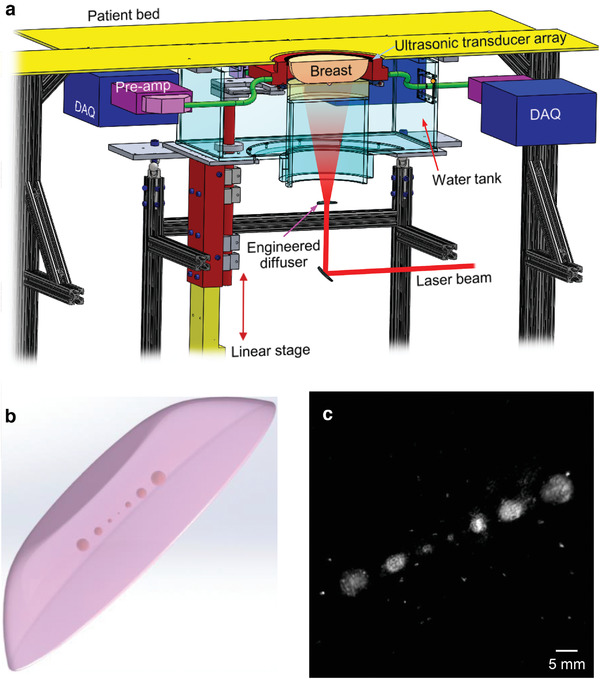
Schematic of the SBH‐PACT and breast phantom imaging. a) Perspective cut‐away view of the SBH‐PACT breast imaging system. DAQ, data acquisition module; Pre‐amp, preamplifiers. b) Sketch of the breast‐mimicking phantom. c) Maximum‐amplitude‐projection (MAP) of the breast phantom image acquired by SBH‐PACT, which revealed the smallest tumor phantom (1‐mm diameter). The bright dots in the image background were caused by air bubbles embedded in the phantom.

As shown in **Figure** **1**a, a patient bed is placed above the SBH‐PACT system with minimal separation (Figure S1, Supporting Information). Laser light is expanded into a homogenized circular beam and directed upward to illuminate the breast that is slightly compressed against the chest wall. A full‐ring ultrasonic transducer array with an in‐plane panoramic view detects photoacoustic waves around the breast. A linear stage motorizes the transducer array to scan the breast elevationally. By scanning within a single breath hold of 15 s, SBH‐PACT can reveal detailed angiographic structures of the entire breast, visualizing vasculatures down to an apparent vascular diameter of 258 µm.^[^ ^23^ ^]^

Before imaging breast cancer patients, we first assessed the system's sensitivity to detecting small abnormalities in the breast by imaging a breast‐mimicking phantom (Figure 1b), which consisted of clear agarose (3%), black acrylic ink (0.0125%), and 20% intralipid (3.6%).^[^ ^33^ ^]^ We used 3D‐printed molds to make the phantom with embedded tumors (Figure S2, Supporting Information). Seven tumor phantoms were embedded in the breast phantom at a depth of 2 cm. The diameters of the tumors were designed to be 1, 1.5, 2, 2.5, 3, and 3.5 mm respectively. The absorption coefficient of the tumors (*μ*_a_ = 0.105 cm^−1^ ) was 2.1 times that of the breast phantom (*μ*_*a*_ = 0.05 cm^−1^ ).^[^ ^20^, ^34^ ^]^ The reduced scattering coefficient $\mu_{s}^{\prime }$ of the phantoms was 5 cm^−1^ at 1064 nm.^[^ ^20^ ^]^ In the phantom image (Figure 1c), the tumors near the side appear larger than what we designed, possibly due to diffusion of the ink. However, the system is sufficiently sensitive to detect a 1 mm‐diameter tumor (near the center), which appears to be the correct size.

### 2.2 Pilot Clinical SBH‐PACT Studies with Patient Reported Outcomes

The primary objectives of this study are to assess the use of SBH‐PACT in women with breast cancer treated with NAC and to develop quantitative measurements to detect responses in the NAC‐treated breast cancers. Four patients diagnosed with breast cancer requiring NAC consented and accrued to the clinical study. The clinical study was closed after the first visit of the fourth patient due to the coronavirus pandemic (Table S1, Supporting Information). However, prior to the study closure, we completed nine SBH‐PACT imaging sessions in three patients (*n* = 3). The patients were imaged at three time points (Figure S3, Supporting Information): (T1) prior to initiation of NAC; (T2) during NAC; and (T3) after completion of NAC and before surgery. The images were compared with other diagnostic imaging acquired as part of standard clinical care (such as mammogram and MRI) as well as with the final histopathological report of the resected breast tissue. All SBH‐PACT images were reconstructed and processed without prior knowledge of clinical images or pathological response.

At the end of each session, the patient completed a self‐administered survey developed to assess the user experience of the SBH‐PACT (Figure S4, Supporting Information). The patients reported that SBH‐PACT was easier than MRI and mammography and comparable to breast ultrasonography. Among the three patients that completed three visits, two patients had incomplete response with solid masses left after the NAC as palpated on clinical exam or imaged on MRI. The other patient had complete clinical response with no definable mass on physical exam and on MRI.

### 2.3 SBH‐PACT of Breast Cancer Treated with NAC

**Figure 2:**
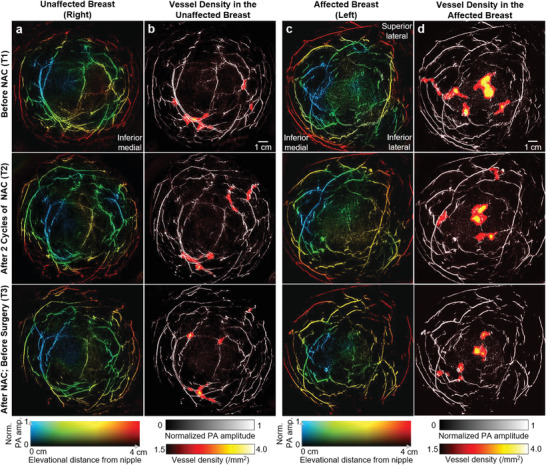
Breast images of a patient treated with NAC. a) Depth‐encoded angiograms of the unaffected (right) breast acquired by SBH‐PACT at three time points. b) Vessel density maps overlaid on the maximum‐amplitude‐projections (MAPs) of the breast images in (a). c) Depth‐encoded angiograms of the affected (left) breast of the patient. d) Vessel density maps overlaid on the MAPs of the images in (c). Regions with higher vascular densities are highlighted.

**Figure** **2** shows SBH‐PACT breast images of one patient at T1, T2, and T3 time points. We listed the color‐encoded depth‐resolved angiograms of the unaffected (Figure 2a) and affected breasts (Figure 2c). The contralateral images of the unaffected breast serve as controls in which angiographic structures were not significantly changed by the NAC.

The cancer appears as a region with higher density blood vessels on the SBH‐PACT images. To automatically highlight such regions in the breast, we extracted the vessel skeletons and generated vessel density maps for each image (Figure 2b,d). The regions with the highest vessel density correspond to the location of the cancer (Figure 2d). The images clearly show the shrinkage of the lesion with NAC although the patient did not have a complete response. The regions with higher vessel density outside the pathologically‐confirmed cancer could be false positives, which, however, might indicate early‐state lesions that have not been clinically diagnosed.^[^ ^35^ ^]^

In the unaffected breasts, we also observed areas with increased vessel densities that were unaccounted for in conventional breast imaging (Figure S5, Supporting Information). Given SBH‐PACT's high sensitivity to microvasculature, the areas with abnormally high vessel densities may represent early neoplastic changes in the breast not detectable by current imaging including MRI. Despite advances in breast imaging technology such as CE‐MRI, there remains up to an 11.3% rate of an occult breast cancers, identified only in the resected specimen.^[^ ^36^ ^]^ We anticipate that SBH‐PACT may improve on detection of early and image‐occult breast lesions. However, it remains possible that these areas of increased vessel density may represent artifact or benign changes in the breast. In unaffected breasts, the enhancement of the regions with higher vessel densities can be eliminated by using a higher vessel density threshold (e.g., 2.5 mm^−2^) and/or rejecting the regions smaller than a certain area (e.g., 4 mm^2^).

**Figure 3:**
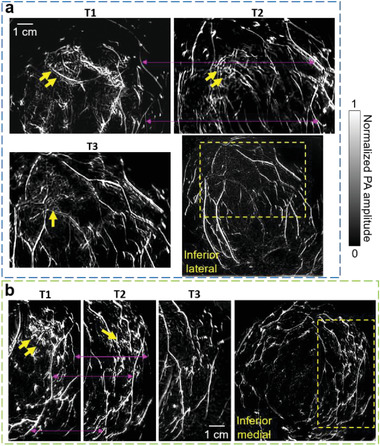
SBH‐PACT images of the other two patients treated with NAC. a) Angiograms of Patient 2 who had a partial response to NAC. Close‐up views of the cancer‐affected area are shown from T1 to T3 in the region outlined by yellow dashed boxes (bottom right corner of figure). Breast cancer is identified by yellow arrows. Prominent common blood vessels in T1 and T2 images are marked by magenta arrow lines. b) Angiograms of Patient 3 who had a complete clinical response to NAC. Angiogenesis associated with the cancer was not detected at T3.

**Figure** **3**a shows another case (Patient 2) with partial response in breast cancer. Angiogenesis near the cancer region was still apparent after NAC at T3. In addition, SBH‐PACT also identified a patient with complete clinical response to NAC (Figure 3b, Patient 3). For this patient, cancer‐associated angiogenesis decreased at T2 and almost disappeared at T3, consistent with the near pathological complete response confirmed by the histopathological examination (Table S2, Supporting Information).

### 2.4 Breast Cancer Segmentation Based on Blood Vascular Irregularity

**Figure 4:**
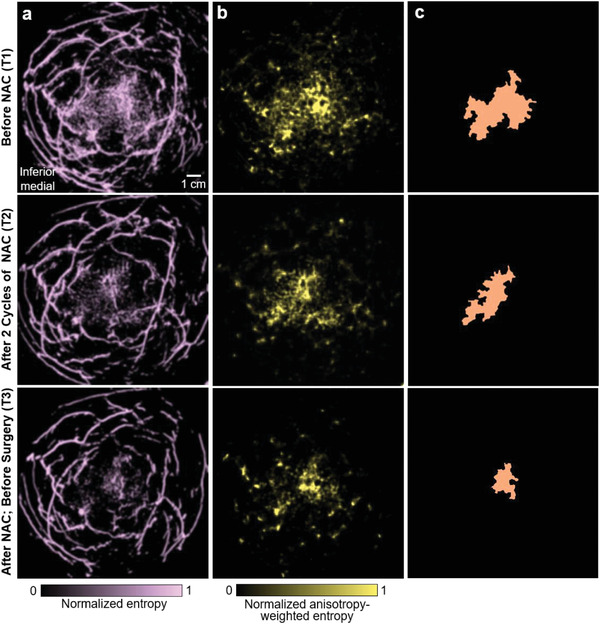
Breast cancer segmentation based on blood vascular irregularity. a) Entropy maps computed from the breast angiograms. b) Anisotropy‐weighted entropy maps, which suppress the structures in healthy tissues. c) Segmented maps of the cancer‐affected area extracted from (b).

In addition to blood vessel density that is observed directly, we also extracted other characteristics of the blood vasculature in the breast. We determined that the cancer‐associated vasculature tend to lack order, i.e., have greater irregularity in distribution and morphology, when compared to healthy breast tissue. To evaluate the randomness of the vascular distribution, we calculated entropy maps of the PACT images (**Figure** **4**a) to quantify PA amplitude fluctuations. Cancers and vessel boundaries have shown higher entropy because cancer‐associated angiogenesis induces more fluctuation than normal breast tissues and blood vessel boundaries have abrupt PA amplitude changes. Since the vasculature in the healthy tissue tends to be smoother and more directional, we further quantified the irregularity of the vascular morphology (i.e., anisotropy) and then used it to weight the entropy map, suppressing the structures in healthy tissues (Figure 4b).

To automatically encircle the breast cancer, we developed an algorithm for cancer segmentation. We binarized the anisotropy‐weighted entropy maps with a threshold of each whole‐breast's average plus 1.4 times the standard deviation. This threshold was chosen so that the segmented tumor sizes were similar to those measured by breast MRI. We then isolated the contiguous region with the largest pixel number to acquire the cancer mask (Figure 4c). Using the cancer masks in SBH‐PACT images, we measured the cancers’ dimensions.

**Figure 5:**
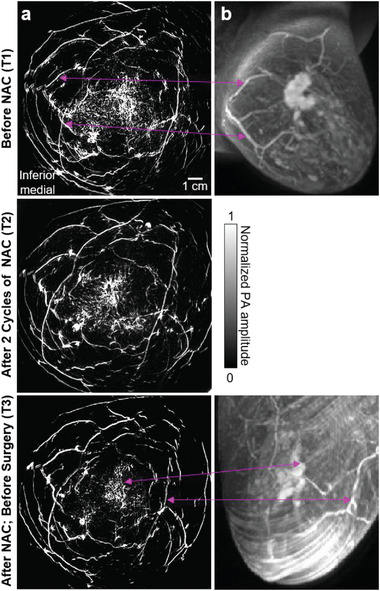
Comparison of the images acquired by SBH‐PACT and MRI. a) SBH‐PACT angiograms weighted by Figure 4b. b) MRI images of the same breast with dynamic post contrast sequence acquired at T1 and T3 of the NAC treatment. Correlated structures are marked by magenta arrow lines. SBH‐PACT reveals more angiographic details within a single breath hold of 15 s.

In addition to cancer segmentation, anisotropy‐weighted entropy maps were also used to modulate the original angiograms (**Figure** **5**a). Since the breasts were compressed differently during PACT and MRI scanning, we rotated the MRI images to a view angle between the craniocaudal view and mediolateral view, so that the relative positions of the tumor and major blood vessels in the PACT images were similar to those in the MRI images. The side‐by‐side comparison of the SBH‐PACT and MRI images at T1 and T3 (Figure 5b) demonstrates concordance of the two imaging modalities in detecting change in the cancer with treatment and the partial response to NAC. However, only the SBH‐PACT images revealed the angiographic details of the response of the cancer; the MRI images did not.

In SBH‐PACT, the spatial resolution in the elevational direction (5.6 mm) is much coarser than the in‐plane resolution (255 µm). Also, the blood vessels in the cancer‐affected areas are close together in the elevational direction (e.g., similar color in Figure 2c). Therefore, we performed quantitative analysis on 2D images that were produced by MAP along the elevational direction. Considering the relatively low blood volume in the breast,^[^ ^37^ ^]^ MAP along the elevational direction may capture most angiographic structures in the breast. It would be more accurate to measure the cancer's characteristics in 3D space, which, however, would require isotropic spatial resolution in 3D space.

### 2.5 Measurements of Breast Cancer Characteristics to Evaluate Response to NAC

**Figure 6:**
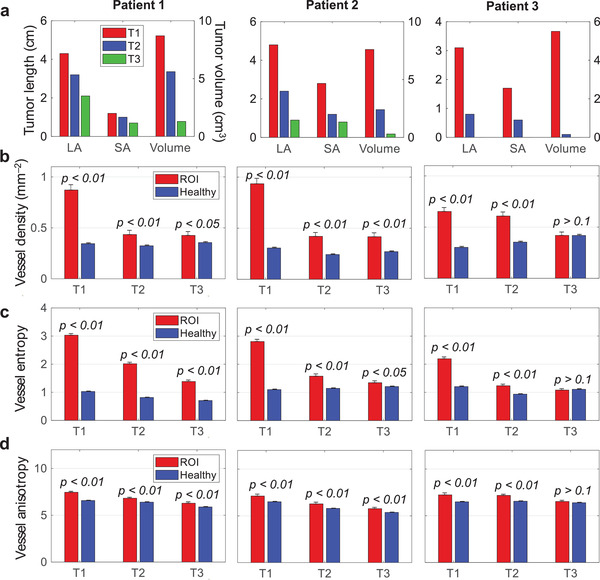
Quantitative measurements of the tumors’ transformation during the NAC. Statistical data are presented as mean ± standard error; *p*‐values are calculated using one‐tailed Welch's (unequal variances) *t*‐test. a) Measurements of tumor dimensions and volume. LA, long axis; SA, short axis. b) Quantification of the relative blood vascular density in the ROI and healthy tissues. c) Quantification of the blood vascular entropy in the ROI and healthy tissues. d) Quantification of the blood vascular anisotropy in the ROI and healthy tissues.

To quantitatively analyze the response of the cancer to NAC, we defined a region of interest (ROI) as the cancer‐affected area in the T1 image and applied the same ROI on the coregistered images of T2 and T3. **Figure** **6** summarizes the cancers’ characteristics we extracted from SBH‐PACT images and used to determine response to NAC. For each image, we quantified the cancer size, blood vessel density, entropy, and anisotropy as measures of the cancer response. The measurements of the cancer dimensions (Figure 6a) in SBH‐PACT images were comparable to those of the clinical imaging modalities (i.e., mammography, ultrasonography, and MRI) and agreed well with the histopathological diagnosis (Table S2, Supporting Information). The smallest cancer identified as a solid mass on SBH‐PACT was 0.8 cm × 0.6 cm × 0.5 cm in Patient 3 at T2. We directly observed the decrease in size of the cancers with NAC in serial SBH‐PACT images of all three patients.

Moreover, we observed noticeable decreases in the relative vascular density (red columns in Figure 6b), entropy (Figure 6c), and anisotropy (Figure 6d) of the cancer with NAC. We imaged the contralateral, unaffected breast of each patient for comparison. The same measurements were almost unchanged with NAC in the unaffected breast (blue columns in Figure 6b–d). To determine if these differences between the affected (ROI) and the healthy breast tissues were significant, we applied a Welch's (unequal variances) *t*‐test to determine the *p*‐values under the null hypothesis that the mean values of parameters in the ROI are not greater than those in the healthy breast tissues. For example, in patient 3, the blood vessel measurements in the ROI and the healthy tissue in the T1‐image were different (*p*‐value < 0.01). However, after NAC, the blood vessel measurements in the T3‐image were not statistically significant, with a *p*‐value > 0.1, suggesting that the patient's breast cancer may have had a complete response to NAC. In comparison, the vessel measurements in the ROI and healthy tissues of Patients 1 and 2 had significant statistical differences (*p* < 0.05) across the T1–T3 images, suggesting the cancers had a partial response to NAC.

The assessment of angiographic microvasculature provided a reliable method to evaluate the anatomic changes of the cancer with treatment. We also calculated the averaged PA amplitude within the ROI and the healthy tissue in the same depth (Figure S6, Supporting Information), showing a decrease in the PA amplitude ratio between the ROI and the healthy tissue over the course of treatment. However, only assessing PA amplitude is not reliable for diagnosis. For example, breast deformation during each visit (T1, T2, and T3) affected optical fluence differently, resulting in noticeable PA amplitude changes in the healthy tissue during the treatment.

## 3 Discussion and Conclusion

In this study, we demonstrate the potential for SBH‐PACT as a diagnostic tool to assess breast cancer responses to chemotherapy. SBH‐PACT clearly depicts detailed angiographic structures from the nipple to the chest wall and localizes the breast cancer with the higher blood vessel density. Processing of the breast images based on vascular entropy and anisotropy further enhances the ability to detect differences between normal breast tissue and breast cancer with treatment and over serial images. Based on these findings, we developed an algorithm for breast cancer segmentation and quantified the cancer size, relative blood vessel density, entropy, and anisotropy as measures of response to NAC. In addition, patient experience of this imaging modality was favorable, with feedback suggesting that SBH‐PACT was comfortable and easier than other breast imaging tests.

We completed SBH‐PACT imaging of the affected and unaffected breast in three separate time points in three patients for a total of nine imaging sessions. The clinical study was closed by the Institutional Review Board at Caltech to protect patients from unnecessary exposure during the coronavirus pandemic. However, the aggregated data from the patients suggests that SBH‐PACT is sufficiently sensitive to capture the angiographic changes in breast cancer during treatment.

The current imaging system can be further improved using a laser with a higher pulse energy and repetition rate, which will increase the signal‐to‐noise ratio to reveal greater clarity of structures previously obscured by the background noise. To reduce the breast thickness for light to penetrate from the nipple to the chest wall, our current system compresses the breast using a soft agar pillow. This method could not fully confine the breast's shape during imaging, thus affecting the reproducibility of serial images. To better control for breast tissue deformation, SBH‐PACT can be equipped with breast holding cups made by a thin layer of polymethylpentene (TPX) that has a similar acoustic impedance to the breast tissue.^[^ ^38^ ^]^ In addition to the improvements in the imaging system, post‐processing of the reconstructed images could further improve imaging sensitivity, especially for the detection of microscopic disease.^[^ ^39^, ^40^, ^41^ ^]^ For example, Liu et al. produced high PA imaging sensitivity by using a multiscale vascular enhancement filter^[^ ^40^ ^]^ and deep learning method^[^ ^41^ ^]^ to reveal detailed structures in PA angiograms.

The clear imaging and accurate evaluation of breast tumor‐associated angiogenesis support further development of SBH‐PACT in breast imaging. Angiogenesis plays a central role in breast cancer development, invasion, and metastasis. Until now, measurements of angiogenesis have not been used in imaging likely because of inability to accurately detect microvascular changes. However, our study demonstrates the use of SBH‐PACT vascular measurements to assess response to treatment in breast cancer. In addition, the ability to fully scan each breast within 15 s and without intravenous contrast or ionizing radiation further support ongoing studies to advance this technology for clinical use.

Other potential applications in breast cancer detection and treatment include screening and surveillance. In addition to the diagnosis of response to the NAC in breast cancer patients, imaging and quantitatively measuring the angiographic environment in the breast using SBH‐PACT has the potential to assist as a prognostic tool to determine relapse‐free survival,^[^ ^42^ ^]^ as a real‐time test of response in drug development,^[^ ^43^ ^]^ and as an alternative to invasive biopsies for diagnosis.

## 4 Experimental Section

##### System Construction and Image Formation

In the SBH‐PACT, a 1064‐nm laser beam from an Nd:YAG laser (LPY 7875‐20, Litron Lasers, Ltd.) was first expanded by an engineered diffuser (EDC‐10, RPC Photonics, Inc.) to form a circular light beam. The laser fluence (28 mJ cm^−2^) was within the American National Standards Institutes (ANSI) safety limit for laser exposure (50 mJ cm^−2^ at 1064 nm at a 20‐Hz pulse repetition rate).^[^ ^44^ ^]^ To achieve 2D panoramic acoustic detection, a full‐ring ultrasonic transducer array is employed with 512 elements (Imasonic, Inc.; 220 mm ring diameter; 2.25 MHz central frequency). The ultrasonic transducer array was enclosed in an acrylic water tank for acoustic coupling to the breast. Four sets of 128‐channel preamplifiers (26 dB gain) and data acquisition systems (SonixDAQ, Ultrasonix Medical ULC) were placed around the water tank, amplifying and digitizing the PA signal. The data acquisition systems were configured to record PA signals within 100 µs after each laser pulse excitation.

The universal back‐projection (UBP) algorithm^[^ ^45^ ^]^ was used to reconstruct all images in this work. The ultrasonic transducer array scanned the entire breast from the chest wall to the nipple, back‐projecting the time‐domain PA signals at all elevational scanning steps into the 3D space. SBH‐PACT has an in‐plane resolution of 255 µm and an elevational resolution of 5.6 mm.^[^ ^23^ ^]^ Each volumetric image was first reconstructed with a voxel size of 1 mm in the elevational direction and 0.1  ×  0.1 mm^2^ on the horizontal plane. All the reconstructed images were further batch‐processed to improve contrast. A depth compensation (e^0.6  × depth(cm)^) method was first applied to enhance the PA amplitude in the deep tissue. The compensated images were then denoised using sparse 4D transform‐domain collaborative filtration.^[^ ^46^ ^]^ Hessian‐based Frangi vesselness filtering^[^ ^47^ ^]^ was then applied to the denoised images to enhance the contrast of blood vessels. Finally, the filtered images (self‐normalized) were then added with a weighting factor of 0.35 to the denoised images with a weighting factor of 0.65 and obtained the presented images.

##### Standard Imaging Procedure

The human studies were completed under institutional approval and oversight by both California Institute of Technology (Committee for the Protection of Human Subjects, 18–0785) and City of Hope National Medical Center (Institutional Review Board, 17 315). Breast imaging was performed in a dedicated human imaging room installed with privacy curtains. Enrolled patients who were scheduled to undergo NAC treatment were imaged using SBH‐PACT at 3 time points: (T1) after the standard care work‐up and the percutaneous biopsy, but prior to initiation of neoadjuvant therapy; (T2) during neoadjuvant therapy; and (T3) after completion of neoadjuvant therapy but prior to surgery. The specific timing of the T2 imaging was set after 2 cycles of chemotherapy (≈4–6 weeks after initiation).

Prior to imaging, the imaging bed and the imaging system were thoroughly sanitized using disinfecting wipes (Super Sani‐Cloth germicidal disposable wipe). The bed was covered by examining table paper discarded after each use. During SBH‐PACT imaging, a female study coordinator assisted the patient in a private space enclosed by curtains. Other researchers involved in the clinical studies operated the device outside the private space. The study coordinator covered the patient's nipples with white nipple masks (3M micropore surgical paper tape), used to partially block the light given the higher concentration of pigment at the nipple. The patient was positioned prone, with one breast placed in the water tank through a large aperture in the bedtop. The water tank was filled with preheated clean water at a temperature of 35 °C.

At the end of each imaging session, the patient was asked to complete a self‐administered two‐page survey. The survey required less than 5 min to complete.

##### Measurement of the Relative Blood Vessel Density

To measure relative blood vessel density, blood vessel skeletons were first extracted by generating vessel centerlines^[^ ^23^ ^]^ from the MAP images. The vessel centerlines were broken into independent vessels at junction points. Independent vessels were then removed with lengths less than 3 pixels to reduce noise. To generate the blood vessel density map, a 2 mm  ×  2 mm window was scanned across the entire vessel skeleton image. The vessel density was quantified as the number of vessels in the window divided by the window area. The vessel density of the window area was then assigned to the window's center pixel.^[^ ^23^ ^]^ We name this parameter relative vessel density because the calculated density depends on the window size which, however, has a minor effect on the *p*‐values of the relative vessel density in *t*‐tests.

##### Measurement of the Vascular Entropy and Anisotropy

To mitigate the background noise and single‐pixel artifacts, thresholding was first applied to the MAPs (2D matrix *M*_PA_) of the batch‐processed images. The threshold was selected as the maximum PA amplitude within the selected background (i.e., a region outside the breast). A 1 mm × 1 mm window was then used to scan across every pixel in the image. For each image subset $M_{\mathrm{PA}}^{\mathrm{sub}}$, we calculated the information entropy *H* within the window

(1) $$H\;\left(M_{\mathrm{PA}}^{\mathrm{sub}}\right)=\;-\sum_{i\;=\;1}^{n}P_{i}\log_{2}P_{i}$$

where *n* denotes the number of discrete bins in the window, *P_i_* denotes the probability for a pixel to have value fallen in the *i* ^th^ bin. The acquired entropy was then assigned to the center pixel of the window, forming an entropy map *M*_e_ (Figure 3A).

Similarly, to measure the vascular anisotropy, the *M*_PA_ was scanned using the same window size. For each subset $M_{\mathrm{PA}}^{\mathrm{sub}}$, the $M_{\mathrm{PA}}^{\mathrm{sub}}$ was rotated from 0° to 180°, with a step size of 10°. At each rotation angle *θ*, the singular value decomposition (SVD) of the rotated $M_{\mathrm{PA}}^{\mathrm{sub}}$ was calculated, acquiring the normalized SVD dominancy term *N*_SVD_ defined as

(2) $$N_{\mathrm{SVD}}\;\left(\theta \right)=\;\frac{\sum_{11}\left(\theta \right)}{\mathrm{tr}\left[\sum \left(\theta \right)\right]}$$

where ∑(*θ*) denotes the corresponding diagonal singular value matrix and $\sum_{11}\left(\theta \right)$ represents the element in the first column of the first row, and tr[∑(*θ*)] denotes as the trace of matrix ∑(*θ*). After rotating the $M_{\mathrm{PA}}^{\mathrm{sub}}$ over 180°, the directionality *D* was calculated within the window defined as

(3) $$D\;=\max_{\theta }N_{\mathrm{SVD}}\left(\theta \right)\;-\min_{\theta }N_{\mathrm{SVD}}\left(\theta \right)$$

The smooth and straight blood vessels in the image tend to have a higher directionality value. To make the measurement more intuitively meaningful, anisotropy as $A\;=\frac{1}{D+\varepsilon }$ was further defined, where *ε* ≈ 0.01 is the regularization term to bound the reciprocal. In each window, the entropy *H* was weighed with *A* to suppress the blood vasculatures in the healthy tissue

(4) $$E=H\cdot \exp\left(-k\cdot A^{-1}\right)$$

where *E* is the anisotropy‐weighted entropy. The coefficient *k* was set to 30. Similarly, *E* was assigned within each window to the window's center pixel, generating the anisotropy‐weighted entropy map *M*_ae_ (Figure 3B).

An automatic tumor segmentation algorithm was developed to assist evaluating the tumors’ changes during the NAC treatment. Thresholding was first applied to *M*_ae_ and then binarized it. The contiguous region was then isolated with the largest area to segment the tumor‐affected region (e.g., Figure 3C).

In addition, the MAP image *M*_PA_ was modulated with *M*_ae_ to acquire image *M*_f_ (Figure 3D) with enhanced lesions and detailed angiographic structures

(5) $$M_{f}=\left(M_{\mathrm{ae}}+\beta \right)\times M_{\mathrm{PA}}$$

where *β* ≈ 0.07 is a background constant to maintain the information in the healthy tissue.

##### Standard of Care Work‐Up, Neoadjuvant Chemotherapy, and Pathologic Diagnosis

All the human experiments followed protocols approved by the Institutional Review Boards of California Institute of Technology (Protocol 18–0785) and City of Hope National Medical Center (Protocol 17 315). Patients for the clinical study were identified and referred by treating oncologists at the City of Hope National Comprehensive Cancer Center. Diagnosis of the breast cancer and work up included combinations of mammography, ultrasound, MRI, and breast biopsies. The decision to treat with NAC and the regimen used was at the discretion of the medical oncologist and based on current treatment guidelines. The post‐NAC imaging and definitive surgery were decided by the physicians based on patient preference, response to therapy, and projected cancer outcomes. SBH‐PACT was performed in a dedicated human breast imaging room at Caltech. After verbally agreeing to participate in the study, the patient signed the informed consent form, formally verifying her understanding of the experiment and agreement to participate. Each patient underwent three SBH‐PACT sessions. At the end of each imaging session, a survey was completed by the patient. All cases were reviewed following receipt of the final pathology report to determine imaging‐pathologic correlation.

##### Statistical Analysis

To examine the significance of difference between the healthy tissue and the tumors, a one‐tailed Welch's (unequal variances) *t*‐test was performed on the healthy and cancerous areas as selected by the binary masks. A sliding window was further applied for relative vessel density, entropy, and anisotropy calculations. The sliding windos have no overlap to ensure numerical indepenence for statistical analysis.

The mean value ± standard error of each image is shown in Figure 6, and the null hypothesis *H*_0_ for each *t*‐test is that the tumor region has less or equal population means as the corresponding healthy tissues. The results for the one‐tailed tests are shown in Figure 6 as *p*‐values. Accordingly, the *p*‐values are generally less than 0.05, indicating a rejection of *H*_0_ at the 5% significance level. In contrast, the *p*‐values for Patient 3 visit 3 were typically larger than 0.1. All statistical analysis was done based on the 2D maps via MATLAB function “ttest2.”

## Conflict of Interest

L.V.W. has a financial interest in Microphotoacoustics, Inc., CalPACT, LLC, and Union Photoacoustic Technologies, Ltd., which, however, did not support this work.

## Supporting information

Supporting Information
