# Supplementary material for: Photoacoustic Computed Tomography of Breast Cancer in Response to Neoadjuvant Chemotherapy
Source: Adv Sci (Weinh). 2021 Feb 23;8(7):2003396. doi: 10.1002/advs.202003396 (PMC8025032; doi:10.1002/advs.202003396)
Supplement: Supplementary file 1 — Supporting Information [file ADVS-8-2003396-s001.pdf]

© 2021 Wiley-VCH GmbH

ADVANCED SCIENCE
Open Access

Supporting Information

for *Adv. Sci.*, DOI: 10.1002/advs.202003396

Photoacoustic Computed Tomography of Breast Cancer in Response to Neoadjuvant Chemotherapy

Li Lin, Xin Tong, Peng Hu, Marta Invernizzi, Lily Lai*, and Lihong V. Wang*

Photoacoustic Computed Tomography of Breast Cancer in Response to Neoadjuvant Chemotherapy

Li Lin[1,†], Xin Tong[1,†], Peng Hu[1], Marta Invernizzi[2], Lily Lai[2,*], and Lihong V. Wang[1,*]

[1] Caltech Optical Imaging Laboratory, Andrew and Peggy Cherng Department of Medical Engineering, California Institute of Technology, Pasadena, CA 91125

[2] Division of Surgical Oncology, Department of Surgery, City of Hope Comprehensive Cancer Center, Duarte, CA 91010

[†] L.L. and X.T. contributed equally to this work.

* To whom correspondence should be addressed. E-mail: llai@coh.org, lvw@caltech.edu

Supplementary Figures

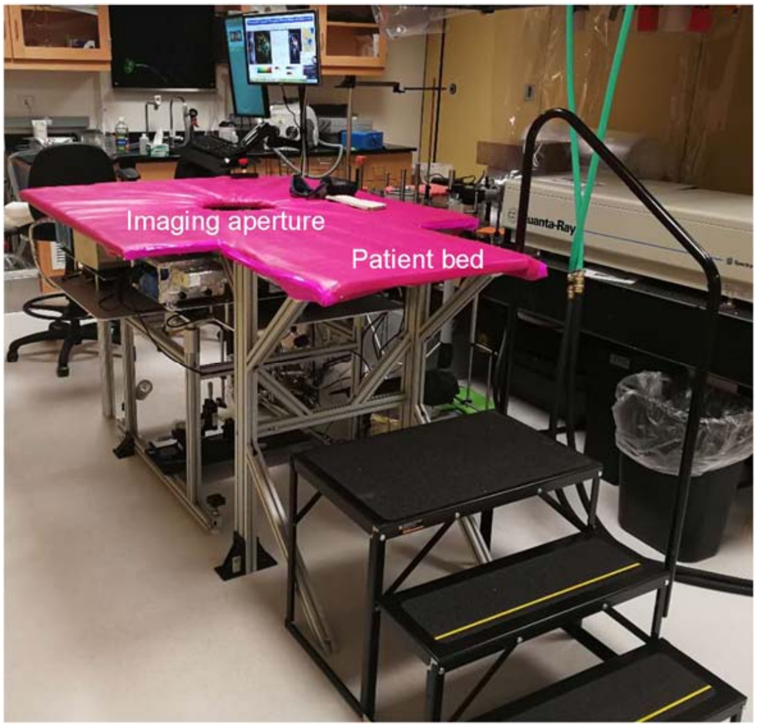

Figure S1. Photograph of the breast imaging room. The patient bed is covered with memory foam and artificial leather. The SBH-PACT system is placed underneath the patient bed. Laser light is guided to the mirror beneath the bed and then reflected upward to the breast.

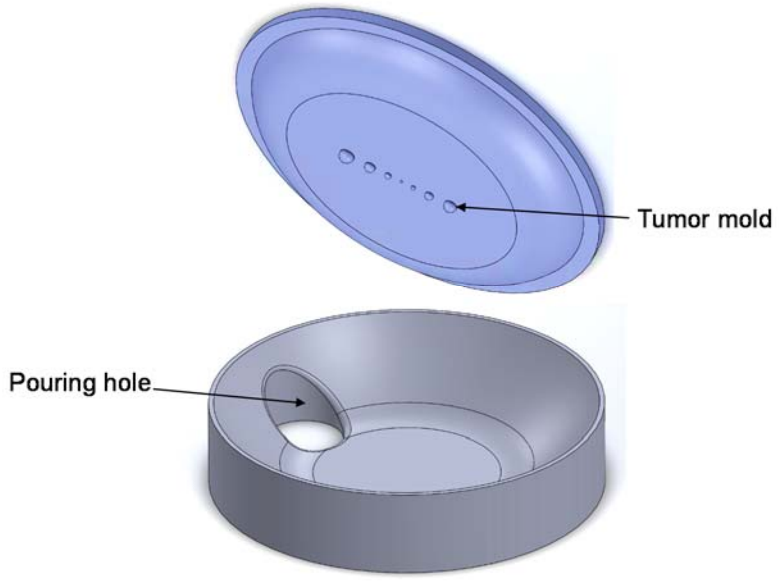

Figure S2. 3D-printed molds used to make the breast-mimicking phantom with tumors embedded.

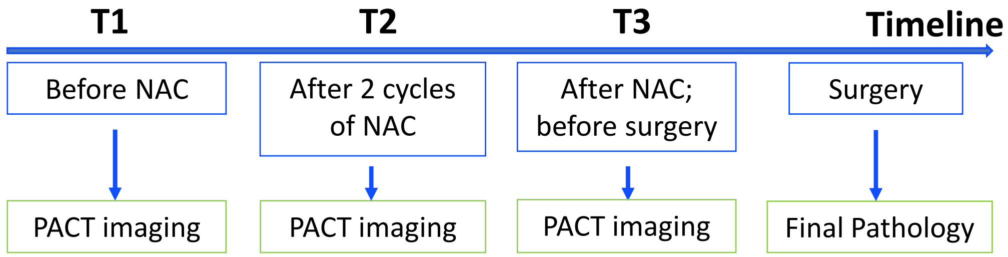

Figure S3. Timeline of the clinical study. PACT images were acquired without viewing clinical images or having the knowledge of pathological results.

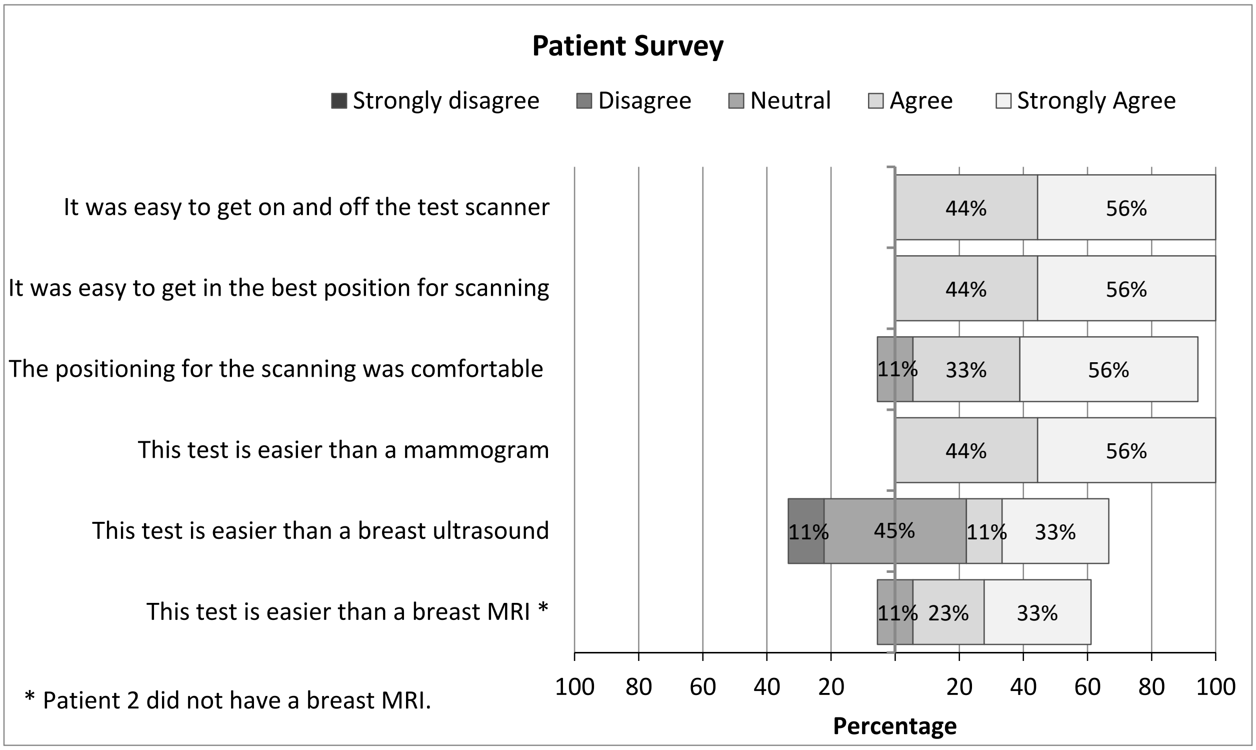

Figure S4. Patients' experience survey. SBH-PACT imaging was easier than mammography and MRI and comparably easy as ultrasonography.

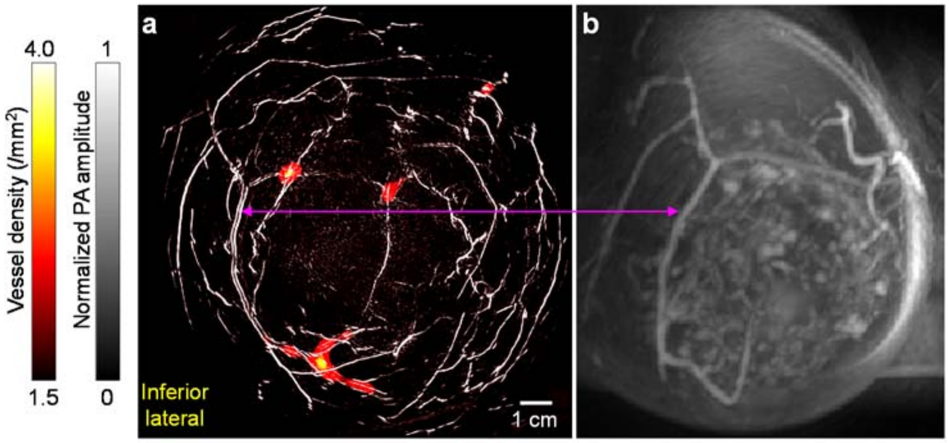

Figure S5. Images of an unaffected breast acquired by SBH-PACT and MRI. a) PACT image with vessel density maps overlaid. b) MRI image of the same breast with dynamic post contrast sequence. Correlated structures are marked by magenta arrow lines.

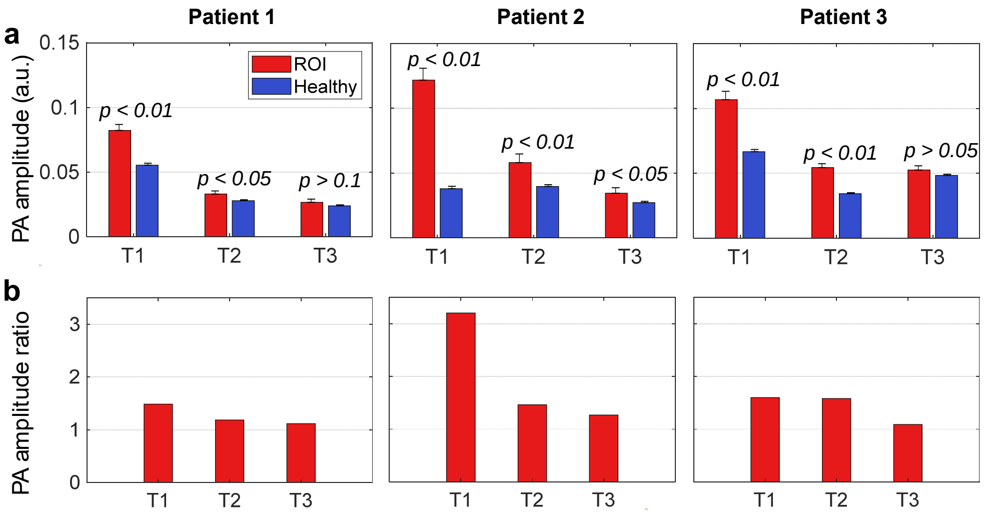

Figure S6. Quantitative measurements of the PA amplitude in breast images during the NAC treatment. a) The PA amplitude in the diseased tissue (ROI) and surrounding healthy tissue was measured in the same depth. b) Ratio of the averaged PA amplitude in the ROI and the healthy tissue.

Supplementary Table

Table S1. Patient information. ER, estrogen receptor; PR, progesterone receptor; HER, hormone receptor; IHC, immunohistochemistry.

Patient info.	Age	Breast size	Affected breast	Clinical stage	Chemo agents	Receptors
**Patient 1**	64	34 D	Left	T3N1	• Adriamycin<br>• Cytoxan	• ER-<br>• PR-<br>• HER-2/neu negative disease
**Patient 2**	39	34 C	Right	IIIB	• Adriamycin<br>• Cytoxan	• ER-<br>• PR-<br>• HER2-
**Patient 3**	50	34 B	Left	T2N2	• Adriamycin<br>• Cytoxan	• ER+ (greater than 95%, 3+)<br>• PR+ (30%, 2-3+)<br>• Her-2/neu by IHC: 1+ (negative for over-expression)<br>• Ki-67: 50%

Table S2. Measurements of tumors' dimensions in SBH-PACT images were comparable to those of the clinical imaging modalities (i.e., mammography, ultrasonography, and MRI) and agreed well with the pathological results. IDC, invasive ductal carcinoma.

Comparison	T1		T2		T3		
**Patient 1**	PACT	MRI	PACT	Palpation	PACT	MRI	Pathology
	4.3 X 1.6 X 2.3 cm	5.0 X 1.8 X 2.5 cm	3.5 X 1.0 X 2.1 cm	1.5 X 0.5 cm	2.1 X 0.8 X 1.2 cm	1.9 X 0.9 cm	Scattered IDC up to 1.1 cm
**Patient 2**	PACT	Mammography	PACT	Palpation	PACT	ultrasonography	Pathology
	2.8 X 4.8 X 1.5 cm	3.6 X 4.5 X 2.0 cm	1.2 X 2.4 X 0.9 cm	1.5 X 1.5 cm	0.8 X 0.9 X 0.5 cm	2.4 X 2.6 X 1.7cm	Focal IDC 0.4 cm
**Patient 3**	PACT	Mammography	PACT	Palpation	PACT	MRI	Pathology
	3.1 X 1.7 X 1.1 cm	3.4 X 1.5 X 1.0 cm	0.8 X 0.6 X 0.5 cm	0 X 0 cm	0 X 0 X 0 cm	0 X 0 cm	Scattered IDC up to 0.1 cm
